# Wild grapes of Armenia: unexplored source of genetic diversity and disease resistance

**DOI:** 10.3389/fpls.2023.1276764

**Published:** 2023-12-08

**Authors:** Kristine Margaryan, Reinhard Töpfer, Boris Gasparyan, Arsen Arakelyan, Oliver Trapp, Franco Röckel, Erika Maul

**Affiliations:** ^1^ Research Group of Plant Genomics, Institute of Molecular Biology of National Academy of Sciences Republic of Armenia (RA), Yerevan, Armenia; ^2^ Department of Genetics and Cytology, Yerevan State University, Yerevan, Armenia; ^3^ Julius Kuehn-Institute (JKI), Institute for Grapevine Breeding Geilweilerhof, Siebeldingen, Germany; ^4^ Institute of Archaeology and Ethnography, National Academy of Sciences Republic of Armenia (RA), Yerevan, Armenia

**Keywords:** wild grapevine, genetic diversity, SSR marker, resistance, Armenia

## Abstract

The present study is the first in-depth research evaluating the genetic diversity and potential resistance of Armenian wild grapes utilizing DNA-based markers to understand the genetic signature of this unexplored germplasm. In the proposed research, five geographical regions with known viticultural history were explored. A total of 148 unique wild genotypes were collected and included in the study with 48 wild individuals previously collected as seed. A total of 24 nSSR markers were utilized to establish a fingerprint database to infer information on the population genetic diversity and structure. Three nSSR markers linked to the *Ren1* locus were analyzed to identify potential resistance against powdery mildew. According to molecular fingerprinting data, the Armenian *V. sylvestris* gene pool conserves a high genetic diversity, displaying 292 different alleles with 12.167 allele per loci. The clustering analyses and diversity parameters supported eight genetic groups with 5.6% admixed proportion. The study of genetic polymorphism at the Ren1 locus revealed that 28 wild genotypes carried three R-alleles and 34 wild genotypes carried two R-alleles associated with PM resistance among analyzed 107 wild individuals. This gene pool richness represents an immense reservoir of under-explored genetic diversity and breeding potential. Therefore, continued survey and research efforts are crucial for the conservation, sustainable management, and utilization of Armenian wild grape resources in the face of emerging challenges in viticulture.

## 1 Introduction

The landscape diversity of Armenia and the peculiarities of the relief are pivotal factors that enrich the plant diversity. Being located in the largely volcanic Armenian Highlands with elevations ranging from 450 to 4,096 m above sea level and situated at the crossroads of two completely different floristic regions, namely, mesophyllous Caucasian and the largely woodless, arid, Armeno-Iranian, gave rise to varied and contrasting environments that support an extremely floristic richness. Armenia is a well-known hotspot of cultivated crop diversity defined by Vavilov (Vavilov, 1926). On a relatively small territory of the country, the flora is represented by approximately 3,800 species of vascular plants from 160 families and 913 genera, including 146 endemic species (Nanagulyan et al., 2020). According to archaeobotanical studies, wheat, barley, rye, oat, pea, melon, watermelon, apricot, pomegranate, and grapes have been cultivated in Armenia since ancient times, and the country is also outstandingly rich in wild relatives of cultivated plants. Crop wild relatives are valuable genetic resources and represent a large pool of genetic diversity for new allelic variation required in breeding programs capable of coping with the major biotic and abiotic stresses (Margaryan et al., 2019).

The cultivated grapevine, *V. vinifera* L. subsp. *sativa* (DC.) Hegi is closely related to, and fully inter-fertile with, an aggregate of wild forms commonly referred to as *V. vinifera* L. subsp. *sylvestris* (C. C. Gmelin) Hegi [= *V. sylvestris* C. C. Gmelin]. In Armenia, *V. vinifera* subsp. *sylvestris* occurs both in the northern and the southern parts of the country, growing in relatively mild subtropical niches in Lori and Tavush provinces, in pre-mountainous areas of Vayots Dzor and Syunik and in floristic regions of Artsakh.

Grapevine has had a unique religious and cultural importance for the Armenians through millennia. In the cuneiform inscriptions of the era of the Van Kingdom, the planting of grapevines, the construction of wine cellars, and other agronomical activities are mentioned. The improvement of viticulture and wine-making traditions in Armenia records significant progress in the Middle Ages, as evidenced by archaeological excavations and bibliographic sources. In the works of Armenian historians and epigraphic inscriptions, there are numerous references to vineyards, wine presses, and wine. The Armenian Church played an important role in the development of viticulture and wine-making as important economic sectors. Almost all monasteries and famous churches had vineyards and wine presses, and wine had an essential role in religious and spiritual life. Grapes were also an important source of food and medicine. Fruit of wild grapevines was harvested to make wine and juice, and the leaves and roots were used for different medicinal purposes. In the book “Haybusak,” the author reports the existence and in detail characterized wild grapes as liana growing in mountains with small, sour, and red berries (Alishan, 1895). In 2007, a quasi-industrial complex for wine production was discovered in Areni-1 cave, built in the limestone rock formations at Arpa River canyon in Vayots Dzor province. Due to the ideal microclimate inside the cave, Areni-1 has yielded large quantities of exceptionally well-preserved organic remains, including grape seeds. Chemical analysis of the excavated material indicates millennia lasting tradition of wine making dating back to 4230–3790 BC. The oldest and best-preserved monument is a testament to the 6,000 years of wine-making tradition in Armenian Highlands (Smith et al., 2014; Hobosyan et al., 2021).

In recent years, in parallel with the wine industry renaissance in the country, only a limited number of traditional varieties were used for wine production with a serious shift to single variety vineyards. The intensive cultivation of a small number of commercial cultivars has resulted in an alarming reduction in genetic diversity, since only 30–35 of 400 native grapevine varieties are used in wine and brandy production. Minor autochthonous cultivars having only a local importance in the different wine-growing regions are under-exploited. Their ignorance might be related to the lack of comprehensive characterization of native neglected varieties, especially to missing data on oenological and agronomical traits and, partially, due to demands of the wine/brandy market. All of these arguments prove the necessity and importance of collection, conservation, characterization, and efficient use of grape germplasm resources, and knowledge of genetic diversity and genetic relationships between grape genotypes. Actually, very little is known about the magnitude of grape germplasm in Armenia. Until now, there have been only a few studies forwarded on characterization of native Armenian grape varieties using molecular-genetic approaches (Dallakyan et al., 2020; Margaryan et al., 2021; Nebish et al., 2021).

Until recently, the exact location of grapevine domestication remains debated. Most lines of evidence point to a primary domestication event in the Near East and Armenian-Persian refuge, but the critical details of grapevine domestication were often inconsistent (McGovern, 2003; Myles et al., 2011; Wan et al., 2013). The large-scale study launched recently elucidates grapevine evolution and domestication history with 3,525 cultivated and wild European grapevines. According to the study, domestication occurred concurrently approximately 11,000 years ago in Western Asia and the Caucasus parallel to yield table and wine grapes, respectively (Dong et al., 2023).

During domestication, essential morphological shifts occurred including larger berry and bunch sizes, higher content of sugar, and altered seed morphology (Zhou et al., 2017). Domestication resulted in most drastic changes in the reproductive biology of the grapevine. Critical were the shift from sexual reproduction to vegetative propagation and the change from a dioecious into a hermaphroditic crop, which is able to pollinate itself and thus set fruit without the need for cross-pollination, leading to bottle necks, limiting genetic diversity. In contrast, dioecious *V. vinifera* subsp. *sylvestris* maintained considerable genetic polymorphism and manifest wide variability. Consequently, seedlings raised from mother plants segregate widely in numerous traits, including size, shape, color, juiciness, sweetness, and palatability of the grape berries (Zohary et al., 2012).

Genetic diversity of wild species is threatened by genetic erosion and extinction due to urbanization, desertification, drought, agricultural development, habitat destruction by overgrazing and forest clearing, and the negative impact of climate change worldwide (Lala et al., 2018; Giovino et al., 2022). *V. vinifera* subsp. *sylvestris* is a unique and valuable genetic resource for the improvement of cultivars in terms of the wide range of tolerance and resistance against biotic and abiotic factors and the high level of adaptation potential in the context of global climate changes (Ocete et al., 1995; Arnold et al., 1998). Thus, the conservation of the existing genetic diversity of wild grapes is essential to safeguard the potential of wild germplasm to be used in future breeding programs. Domestication is an evolutionary process where strong selection for specific traits, combined with population bottlenecks, greatly alter the genetic structure of populations and the underlying genetic architecture of phenotypic traits (Pusadee et al., 2009). Loss of genetic diversity has profound implications for crop improvement. Therefore, potentially valuable traits may be lost in vine cultivars, thereby reducing the set of phenotypes that can, later on, be used by breeders for variety improvement. In this context, the frequent insufficiency of new traits in grapevine germplasm collections has led to increased efforts to preserve wild relatives of domesticated plants, as a reservoir of phenotypes for future crop improvement.

The richness of extensive natural biodiversity of *V. vinifera* L. in Armenia is an unexplored source with great potential to regulate undesirable pests and diseases. The first records pointed to the potential of wild grapes done by Sosnovskiy (1947), who mentioned resistance of *V. sylvestris* from Lori against powdery mildew by observing plants for many years in the forests in natural conditions (Sosnovskiy, 1947). He observed also that these plants might be tolerant against *Phylloxera*. However, during the Soviet Era, breeding programs, even being quite active, have been focused mainly on creation of table grapes and bred cultivars for brandy and vodka production. It is only in 2017 in the frame of Armenian-German bilateral cooperation that the large-scale research was initiated focusing on comprehensive characterization of grapevine genetic resources of Armenia. In their research, Margaryan et al. (2019) described and characterized wild genotypes collected from Syunik province (Margaryan et al., 2019). Authors studied the genetic diversity of wild grapevines concerning their capacity for stilbene biosynthesis, since phytoalexins, as the stilbenes, are the central element of basal immunity, which might be exploited as a genetic resource for resistance breeding. Among analyzed wild individuals, potential genotypes have been selected for future studies.

The study of Riaz et al. (2020) confirmed the resistant potential of Armenian wild grapes against powdery mildew. The authors discovered and characterized an additional gene pool that shared a Ren1-like local haplotype. The results endorse the hypothesis that mildew resistance was present in wild plants and potentially evolved through sexual recombination. Authors suggested a notion that wild progenitor *V. sylvestris* may have developed PM resistance over a long time. It is also possible that resistance was introduced into cultivated *V. vinifera* ssp. *sativa* in certain regions at the time of domestication. Being selected, the Ren1 haplotype stayed intact in *V. vinifera* ssp. *sativa* accessions due to the practice of clonal propagation (Riaz et al., 2020). Recently, Possamai et al. (2021) described the Ren1.2 loci, a new variant of Ren1, was located in the same chromosomal region as Ren1. The loci mapped in the Georgean variety ‘Shavtsitka’, which were classified as a variant of the previously mentioned locus. Both the loci Ren1 and Ren1.2 were found in cultivars of different origins, proposing that Caucasus grapevines have independently developed their resistance loci in the exact same location as Ren1. Based on results provided by authors, the Ren1.2 locus shows partial resistance to *E. necator*, reducing hyphal proliferation and sporulation (Possamai et al., 2021).

Recent studies, strongly increase the interest in Caucasian grape germplasm not only for the richness of genetic diversity but also for the breeding resistant grape varieties and have shown that the resistance trait appears to be widely diffused in such germplasm (Possamai et al., 2021; Vezzulli et al., 2022).

Hence, the aim of the present study was to analyze genetic diversity and structure among Armenian wild grapevine populations prospected from the different regions of the subspecies’ range in Armenia to explore the level of genetic signature of this germplasm and its genetic pattern. The deeper study of Armenian grape germplasm required to uncover further source of resistance. Obtained results will guarantee baseline information for the development of suitable conservation strategies for better management and maintenance of wild grapevine populations in Armenia.

## 2 Materials and methods

### 2.1 Plant material

**Figure 1:**
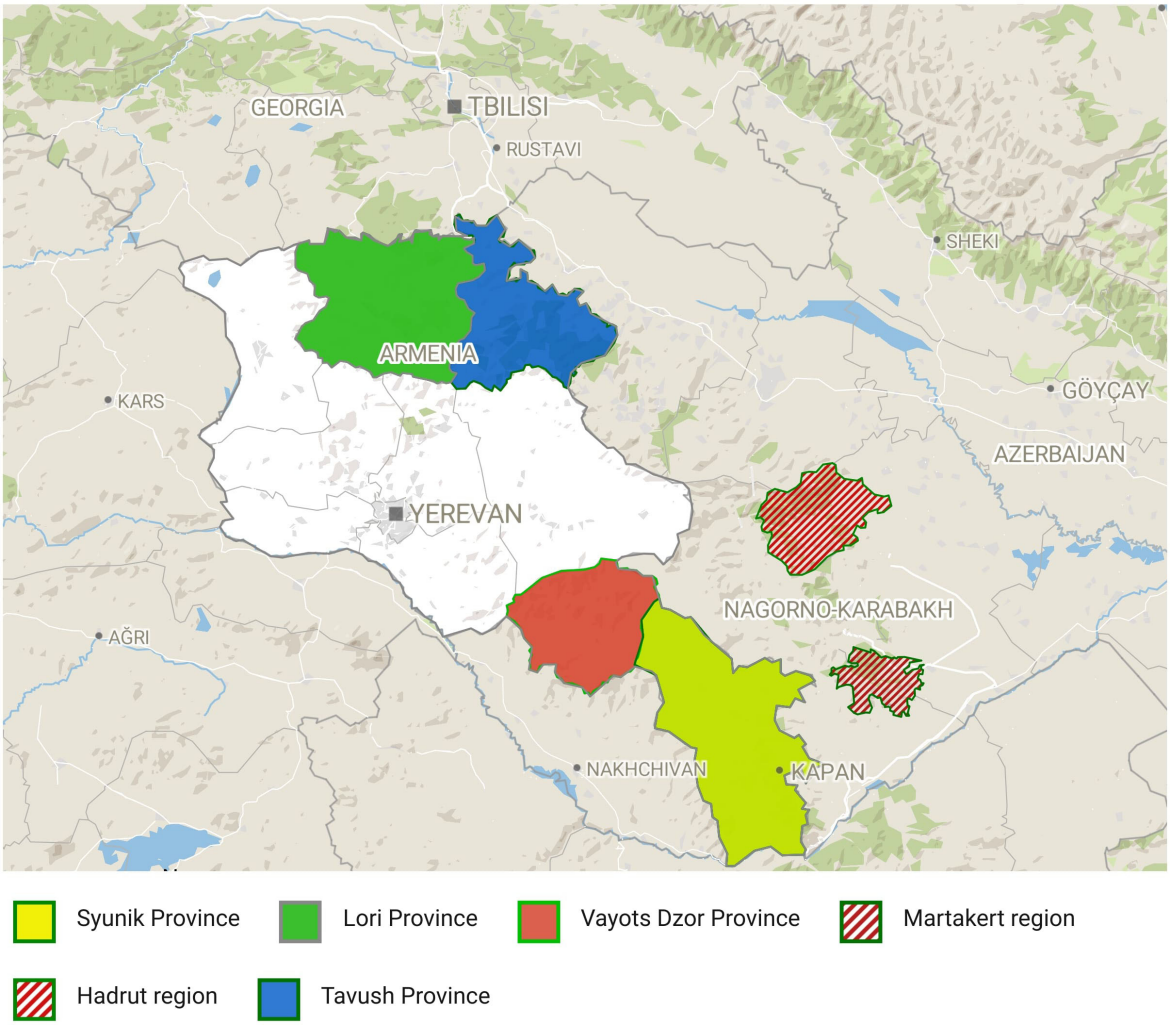
Geographic distribution of the sampled *V. vinifera* L. subsp. *sylvestris* populations in Armenia and Artsakh (Nagorno Karabakh).

The present study on natural populations of wild grapes started by investigating historical bibliography and existing records about the presence of wild grapes in the territory of Armenia and Artsakh located at the northeastern end of the Armenian Highlands in the Lesser Caucasus mountain system. Wild *Vitis* germplasm was collected from the following four administrative regions of Armenia (Syunik, southernmost province; Vayots Dzor, southeastern province; Tavush, northeast province and Lori, north province) during surveys between 2018 and 2022 in their natural habitat: main mountainous areas, climbing the rocks, and embracing the trees, riverbanks, and forests (**Figure 1**). During material sampling, one of the important criteria was to collect wild grapes from isolated areas, located as far as possible from vineyards and home gardens (the nearest sampling area was 10–15 km from vineyards, and in the Syunik region, the material was collected from areas approximately 40–50 km distance from villages). Considering the phenotypic similarity of wild and cultivated grapevines, the wild grapevine sampling strategy for each selected *V. sylvestris* candidate was based on the main differentiating reference traits to distinguish wild from domesticated grapevines in order to reduce as much as possible the risk of collecting plants derived from hybridization with a cultivated grapevine. Each selected sample was analyzed ampelographically, and only samples that met the basic phenotypic profile of wild grapes were subjected to further genetic analysis. During surveys, the following OIV descriptors were used: OIV151, OIV 001, OIV 202, OIV 204, OIV 223, and OIV 225.

A total of 148 unique *V. sylvestris* genotypes were included and analyzed in this study using 24 nSSR markers. Initially, 220 putative wild individuals were collected. The excluded accessions were redundant genotypes, accessions with more than 40% missing microsatellite data, escaped cultivars from vineyards, and feral types. To identify feral types, we performed a parentage analysis between Armenian *V. sylvestris* and autochthonous cultivated accessions; the dataset from Caucasian wild and cultivated grapes also was used (unpublished data). Cervus 3.0.7 was applied as one of the approaches to screen the material (Margaryan et al., 2021).

Among the analyzed set, 61 samples were collected from pre-mountainous areas of Syunik province, 46 samples from Artsakh, 15 samples from Vayots Dzor province, 15 samples from Tavush province, and 11 samples from Lori. GPS coordinates and habitat notes were recorded when the samples were collected from their natural habitats. A part of the accessions was also photo documented to aid with subspecies identification. Young leaves were collected from actively growing shoots, placed in filter paper, air-dried, and stored in paper envelopes for DNA extraction and further use.

Additionally, a subset of 48 wild genotypes collected in the beginning of 2000s and analyzed by Riaz et al., 2018 were added to the study to discover their relationship to the set of recently collected material (Riaz et al., 2018). According to the information provided by authors, these 48 wild accessions were collected from Alaverdi (Lori province) as seeds from female vines. Seedling plants from seed lots were maintained in the USDA National Clonal Germplasm Repository in Davis, CA, USA. Thus, the following analysis will generate results from a total of 196 wild genotypes. For 48 seedlings, nSSR marker data have been extracted and adapted. To distinguish the two data sources, the 148 samples collected for the present study and the 48 seedlings from Riaz et al. (2018) will be designated as genepool A and genepool B, respectively.

### 2.2 DNA extraction and nSSR analysis

Total genomic DNA was extracted from dried leaf tissue after grinding with MM 300 Mixer Mill system (Retsch, Haan, Germany). DNA extraction was performed, employing the DNeasy 96 plant mini kit (QIAGEN, Düsseldorf, Germany) following the manufacturer’s protocol. DNA concentration and quality were checked by spectrophotometric analysis and electrophoresis in 1% agarose gel. Microsatellite fingerprinting of genotypes was performed on 24 microsatellite loci (nSSRs) well distributed across the 19 grape chromosomes as previously described (Sefc et al., 1999; Laucou et al., 2011) [i.e., VVS2, VVMD5, VVMD7, VVMD21, VVMD24, VVMD25, VVMD28, VVMD27, and VVMD32; four of the VrZAG series (VrZAG62, VrZAG79, VrZAG67, VrZAG83); VMC4f3.1; VMC1b11; and nine of the VVI series VVIb01, VVIn16, VVIh54, VVIn73, VVIp31, VVIp60, VVIv37, VVIv67, and VVIq52]. Nine polymorphic microsatellite markers proposed by the GrapeGen06 project, namely, VVMD5, VVMD7, VVMD25, VVMD27, VVMD28, VVMD32, VVS2, VrZAG62, and VrZAG79, were used for comparison of genetic profiles with the SSR-marker database of the Julius Kühn-Institut (JKI), maintaining approximately 8,000 genetic profiles from distinct sources.

For fragment length determination by capillary electrophoresis on ABI 3130xl Genetic Analyzer (Applied Biosystems, Life Technologies, Waltham, MA, USA), all forward primers were 5′-labeled with a fluorescent dye (FAM, HEX, TAMRA, ROX, and PET). The combination of markers with different labels and diverse fragment lengths allows one to perform the polymerase chain reaction (PCR) and grouped markers in seven multiplex pools, comprising two to five SSR markers characterized by similar annealing temperatures (**Supplementary Table S1**). The 2x KAPA2G Fast PCR Kit (Düren, Germany) was used to set up 5-μL reaction mixtures containing master mix, 100 pmol of each primer, and 1 ng of template DNA. A GeneAmp PCR system 9700 thermal cycler (Applied Biosystems, Schwerte, Germany) was used for the amplification starting with 3 min initial denaturation at 95°C, followed by 30 cycles for 30 s. A final extension was performed at 72°C for 7 min. One microliter of the PCR product was used for fragment length determination, and the results were processed with GeneMapper 5.0 software (Applied Biosystems, Life Technologies, Waltham, MA, USA) recorded in base pairs. Allele size was determined by comparing the fragment peaks with the internal size standard, using the Microsatellite default method for size calling with SSR and the expected repeat size. To correct the amplification shifts among different multiplexes, SSR profiles were adapted by including in each PCR amplification run the DNA of standard cultivars Cabernet franc and Muscat à petits grains blancs.

### 2.3 Flower phenotype analysis

The determination of flower sex was carried out for all genotypes collected throughout Armenia and was analyzed by a specifically designed APT3 marker from adenine phosphoribosyl transferase gene capable to distinguish flower sex: female (F), male (M), or hermaphrodites (H) (Fechter et al., 2012). Field phenotypic screening was done only for part of accessions: 46 accessions from Artsakh, 45 accessions from Syunik, 15 accessions from Vayots Dzor, 12 accessions from Tavush, and 4 accessions from Lori. Because of geographic location and relief, it was not always possible to screen flower phenotypes in natural habitats.

### 2.4 Powdery mildew (*Erysiphe necator*) evaluations

A total of 107 non-redundant wild genotypes from five natural populations in Armenia (*in situ*) were analyzed in this study at the Ren1 locus. PM symptoms were observed on wild individuals *in situ* during an inventory of *V. sylvestris* germplasm using a five-class scale (1–9) OIV 455 descriptor. The natural habitats of the studied species cover diverse geographic origins southeastern province and the pre-mountainous area of Syunik in southern Armenia, Artsakh, in the north-eastern Armenian Highlands, northeast Tavush province, and northern Lori province. The overall health status of each plant was evaluated; symptomatic plants were excluded from the analyzed set. Additional three microsatellite markers, SC47-18, SC8-0071-14, and SC175-1, that are associated with the Ren1 gene for PM resistance were analyzed (Riaz et al., 2013; Riaz et al., 2018; Possamai et al., 2021; Lukšić et al., 2022). The SSR markers were multiplexed within two runs. All forward primers were labeled on the 5′ end with fluorescent dyes (HEX, 6-FAM). The following steps, including mpxPCR and fragment length determination by capillary electrophoresis, were carried out as described above. To correct the amplification shifts among multiplexes, SSR profiles were adapted by including in each PCR amplification run the DNA of Kishmish Vatkana, 2010-007-0027 and Vitis Syl. Geo W31 accessions.

### 2.5 Data analysis

The genetic diversity among groups and over all the groups of wild grapes was estimated. The standardized nSSR genotyping data were used to determine the number of different alleles (Na), the number of effective alleles (Ne), Shannon’s Information Index (I), observed heterozygosity (Ho), expected heterozygosity (He), fixation index (F), also called inbreeding coefficient, and private alleles (PA). The allele frequency for each nSSR locus was calculated as well. GenAlEx software version 6.5 was used to compute genetic diversity statistics for each nSSR locus (Nei, 1973; Peakall and Smouse, 2006).

Clustering was performed by MEGA 7 software, version 7.0.26, which was used to generate a distance tree by the neighbor-joining (N-J) hierarchical clustering method using the codominant genotypic distances between all pairwise combinations calculated by the GenAlEx 6.5 (Saitou and Nei, 1987; Kumar et al., 2016). Principal coordinates analysis (PCoA) was performed by GenAlEx 6.5 via covariance matrix with data standardization (Kalinowski et al., 2007). Analysis of molecular variance (AMOVA) was performed to characterize the partition of the observed genetic variation among and within populations and genetic groups using GenAlEx 6.5 software (Excoffier et al., 1992; Kalinowski et al., 2007). The significance test was performed over 999 permutations.

Bayesian clustering was applied on the 24 nSSR genotype data for the wild distinct genotypes. The admixture model in Structure 2.3.4 was employed to infer the number of genetic populations (K) existing in the samples and to assign genotypes to populations of origin, with no prior information (Pritchard et al., 2000). The Structure configuration was set to ignore population information and use an admixture model with correlated allele frequencies. Various numbers of putative populations (K) were tested, ranging from 1 to 10. Burning time and replication number were set to 100,000 and 100,000, respectively, in each independent run with 10 iterations. The choice of the most likely number of clusters (best K) was evaluated following the *ad hoc* statistic delta K (ΔK) as described Evanno et al. (2005) using Structure Harvester (Evanno et al., 2005; Earl and vonHoldt, 2012). The Structure bar plot was visualized by running the clump file obtained by Structure Harvester, in Structure Plot v 2.0 (Ramasamy et al., 2014).

## 3 Results

### 3.1 Flower characterization

The key trait distinguishing *sativa* vs. *sylvestris* subspecies is the flower sex, since wild grapevine is dioecious, whereas flowers of cultivars are usually hermaphroditic. During our surveys, the search for wild plants was focused on collecting dioecious accessions. For 122 wild individuals, the flower morphology was analyzed *in situ*, and for the whole material, the flower phenotype was analyzed by APT3 marker. Sex expression in *Vitis* flower is thought to be controlled by a major locus with three alleles, male M, hermaphrodite H, and female F, with an M > H > F allelic dominance (Picq et al., 2014). In wild individuals, males are MM and MF and females are FF, and in cultivars, hermaphrodites can be HF or HH. According to the obtained data among 196 *sylvestris*, five different allelic patterns were determined at APT3 loci classifying genotypes as F: 39 genotypes have shown 268/268 (F), 20 genotypes 268/397 (F), 33 genotypes 268/336 (F), 3 genotypes 336/336 (F), and 4 genotypes 268/336/397 (F).

Six different allelic patterns were determined at APT3 loci describing genotypes as M: 37 genotypes 268/466 (MF), 15 genotypes 336/466 (MF), 16 genotypes 268/397/466 (MF), 13 genotypes 268/336/397/466 (MF), 8 genotypes 268/336/466 (MF), and 4 genotypes 466/466 (MM). Thus, among the analyzed samples based on molecular phenotyping, 51.04% were female and 48.95% were male individuals. For four genotypes out of 196, DNA analysis was not applicable. Flower phenotypes estimated by DNA-based flower sex marker and field phenotyping of *V. sylvestris* are presented in **Supplementary Table S2**.

### 3.2 Genetic diversity of wild grapes in Armenia

**Table 1** displays the diversity parameters across all wild accessions for 24 nSSR markers. A total of 292 alleles were detected across all markers with an average of 12.167 alleles across 196 accessions. The number of alleles ranged from 4 (VrZAG83, VVIn16) to 22 (VMC4f3.1). The number of effective alleles ranged from 1.978 (VVIn16) to 9.828 for VMC4f3.1. The highest Shannon’s information index (I) was observed in VMC4f3.1 locus (2.579) and lowest in VVIn16 (0.879), while the average among SSR loci was 1.874. Shannon’s information index is an important parameter mirroring the level of polymorphism.

**Table 1:** Diversity indices calculated for 196 distinct genotypes of wild grapevines determined from 24 nuclear microsatellite data.

Locus	Ra (bp)	Na	Ne	I	Ho	He	F
**VVS2**	125–157	13	7.447	2.189	0.793	0.866	0.084
**VVMD5**	226–248	11	6.017	1.991	0.733	0.834	0.121
**VVMD7**	235–263	13	6.922	2.146	0.799	0.856	0.066
**VVMD25**	237–271	11	3.598	1.522	0.691	0.722	0.043
**VVMD27**	176–198	12	5.548	1.907	0.740	0.820	0.098
**VVMD28**	218–282	18	7.290	2.292	0.790	0.863	0.084
**VVMD32**	240–292	15	6.600	2.157	0.771	0.848	0.092
**VrZAG62**	188–204	8	5.595	1.812	0.850	0.821	−0.035
**VrZAG79**	237–261	12	6.991	2.158	0.830	0.857	0.032
**VVIv67**	348–401	18	5.315	2.095	0.761	0.812	0.063
**VrZAG67**	122–159	14	5.815	2.046	0.797	0.828	0.037
**VrZAG83**	188–201	4	3.692	1.344	0.710	0.729	0.026
**VVIn16**	147–155	4	1.978	0.879	0.443	0.495	0.104
**VVIn73**	258–272	6	2.241	0.974	0.510	0.554	0.078
**VVIp60**	274–331	15	6.661	2.163	0.701	0.850	0.176
**VVMD24**	204–218	8	4.211	1.613	0.747	0.763	0.020
**VVMD21**	244–267	9	4.316	1.619	0.626	0.768	0.186
**VMC4f3.1**	163–217	22	9.828	2.579	0.802	0.898	0.107
**VVIb01**	289–317	11	3.203	1.433	0.660	0.688	0.041
**VVIh54**	139–179	15	5.917	1.991	0.747	0.831	0.101
**VVIq52**	70–86	9	3.305	1.468	0.655	0.697	0.061
**VVIv37**	148-182	13	8.309	2.231	0.758	0.880	0.138
**VMC1b11**	167–203	16	4.947	1.957	0.663	0.798	0.169
**VVIp31**	157–195	15	9.692	2.411	0.864	0.897	0.037
*Total*		*292*					
*Min.*		*4*	*1.978*	*0.879*	*0.443*	*0.495*	*−0.035*
*Max*		*22*	*9.828*	*2.579*	*0.864*	*0.898*	*0.186*
*Mean*		*12.167*	*5.643*	*1.874*	*0.727*	*0.791*	*0.080*

Ra, range of allele size (bp); Na, number of different alleles; Ne, effective alleles; I, Shannon’s information index; Ho, observed heterozygosity; He, expected heterozygosity; F, fixation index.

For microsatellite markers efficiency, the observed and the expected heterozygosity (Ho, He) are considered to evaluate the genetic variability among the samples analyzed. Both observed and expected heterozygosity varied among loci. Observed heterozygosity of the analyzed set was lower than the expected heterozygosity for the majority of the analyzed markers, with a mean value of 0.727 and 0.791, respectively. Lower values of observed heterozygosity in conjunction with the results of the fixation index point to the existence of inbreeding, as F-values are expected to be close to zero in case of random mating. Based on our results, the differences between Ho and He were not significant, pointing to random mating promotion. The locus with the lowest F-value was VrZAG62 (−0.035), while the highest was VVMD21 (0.186). The mean F-value for the dataset was 0.080.

**Figure 2:**
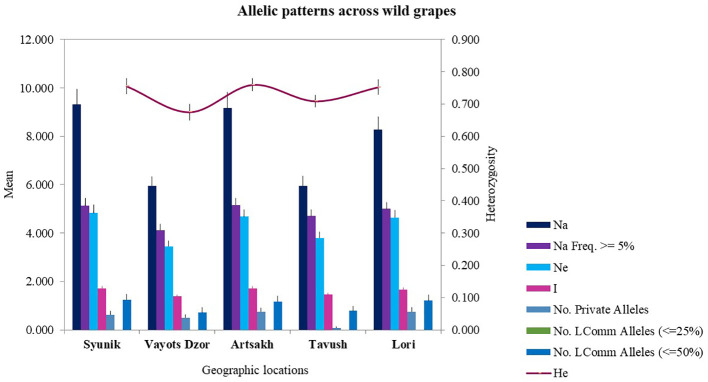
Allelic patterns across wild grapes collected from five geographical locations.

The SSR marker data were divided into five genetic groups/populations based on geographic origin or habitat of collected samples, and the allelic profiles were used to calculate statistical indices to determine diversity within and among the groups (**Figure 2**).

The mean value of number of alleles for all populations is 12.167. The Vayots Dzor and Tavush groups were the least diverse with an average of 5.958 alleles, and the Syunik group was the most diverse with 9.333 alleles. The other two genetic groups (Artsakh and Lori) showed a comparable number of alleles that ranged from 9.167 to 8.292 (**Supplementary Table S9**). The observed heterozygosity (Ho) was lower than the expected heterozygosity (He) for Artsakh, and no difference was observed for the Syunik and Alaverdi groups. For the groups Lori, Tavush, and Vayots Dzor, the expected heterozygosity (He) was slightly higher than the observed heterozygosity (Ho). Expected heterozygosity ranged from 0.569 to 0.760 between the groups. From all groups, the highest He was registered for the Artsakh group with a mean of 0.760, and the lowest He was the Alaverdi group with 0.569.

A total of 65 private alleles were found across all markers within five groups. Private alleles for every group and allelic patterns across *V. sylvestris* are presented in **Supplementary Table S3** and **Figure 2**. The wild genotypes from Lori, Syunik, and Artsakh had by far the most private alleles compared with the other two groups. Overall, statistical indices found these three groups to be the most diverse and Tavush and Vayots Dzor the least diverse. The highest number of private alleles was identified for VMC4f3.1 and VMC1b11 markers in wild populations. Analysis summary of allele size (AS) and frequencies (AF) for each of the microsatellites is presented in **Supplementary Table S4**. Genetic profiles of analyzed non-redundant 196 wild grape genotypes are provided in **Supplementary Table S5**.

### 3.3 Cluster analysis

**Figure 3:**
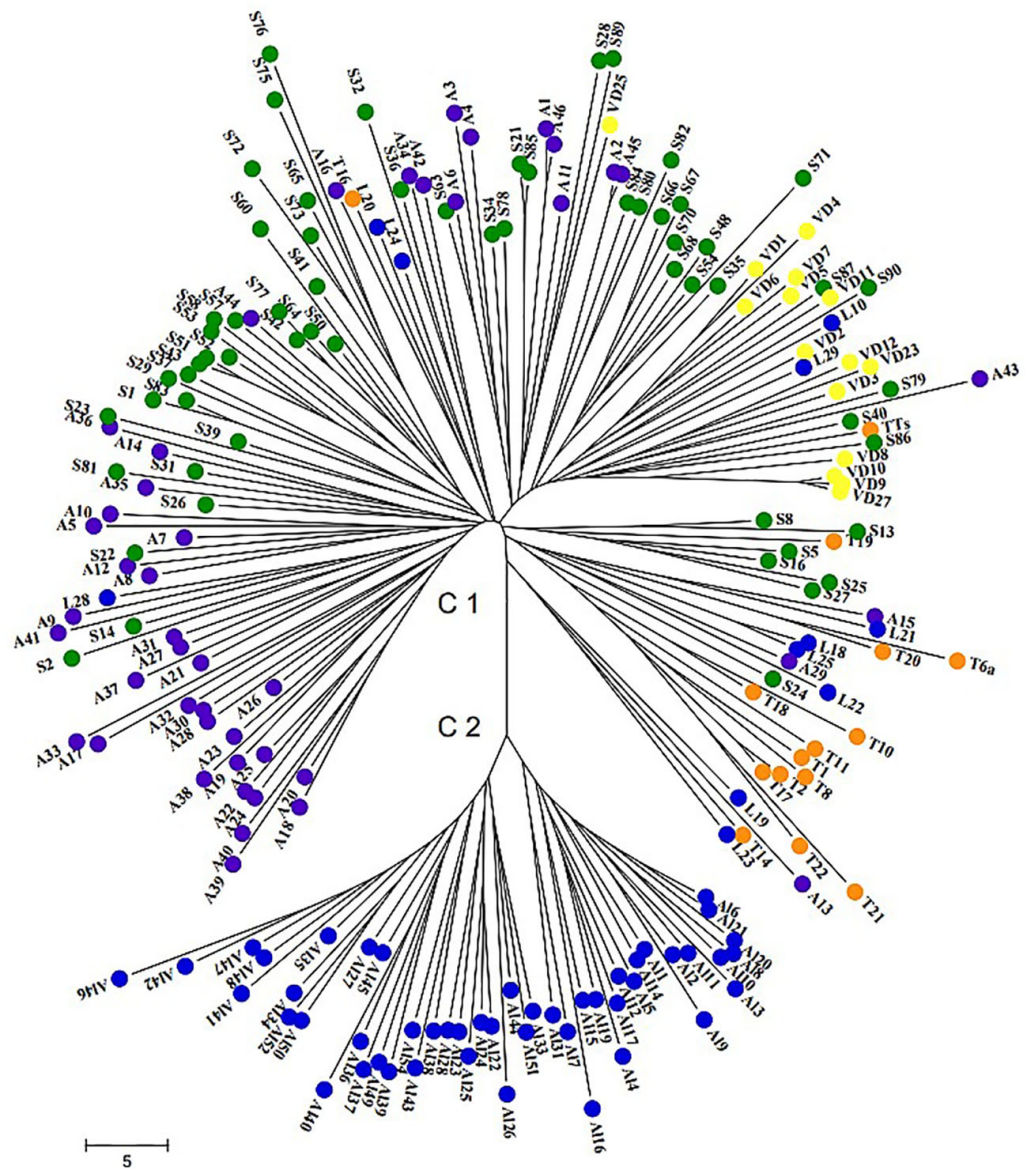
Neighbor-joining dendrogram demonstrating genetic relationships among 196 Armenian wild grape accessions based on 24 nSSR loci. Wild grapes originated from Syunik province are marked in green, wild grapes from Artsakh in violet, wild grapes from Lori province in blue, wild grapes from Tavush province in orange, and wild grapes from Vayots Dzor province in yellow.

The neighbor-joining (NJ) distance tree was constructed to study genetic relationships among 196 wild grape genotypes based on allele frequencies of 24 nSSR loci. Two major clusters with sub-clusters were clearly distinguished. The first cluster contained all samples of genepool A collected in different geographical regions across Armenia and the second cluster grouped genepool B that originated from Lori, Alaverdi, which were seedling plants from seed lots (**Figure 3**). Cluster 2 with two clearly separated sub-clusters is the most divergent clade and formed a separate genepool, not related to genepool A. In this case, we can hypothesize that genotypes involved in genepool B derived from one or closely related mother plants. Thus, the wild individuals of genepool A and genepool B from Lori were not assigned to the same cluster. The geographic clustering as single separate clades can be seen in the *sylvestris* originating from Syunik, Artsakh, Tavush, and Vayots Dzor province, while some of the wild individuals from Lori and Vayots Dzor were represented in blend groups as well.

### 3.4 Population structure analysis and differentiation

**Figure 4:**
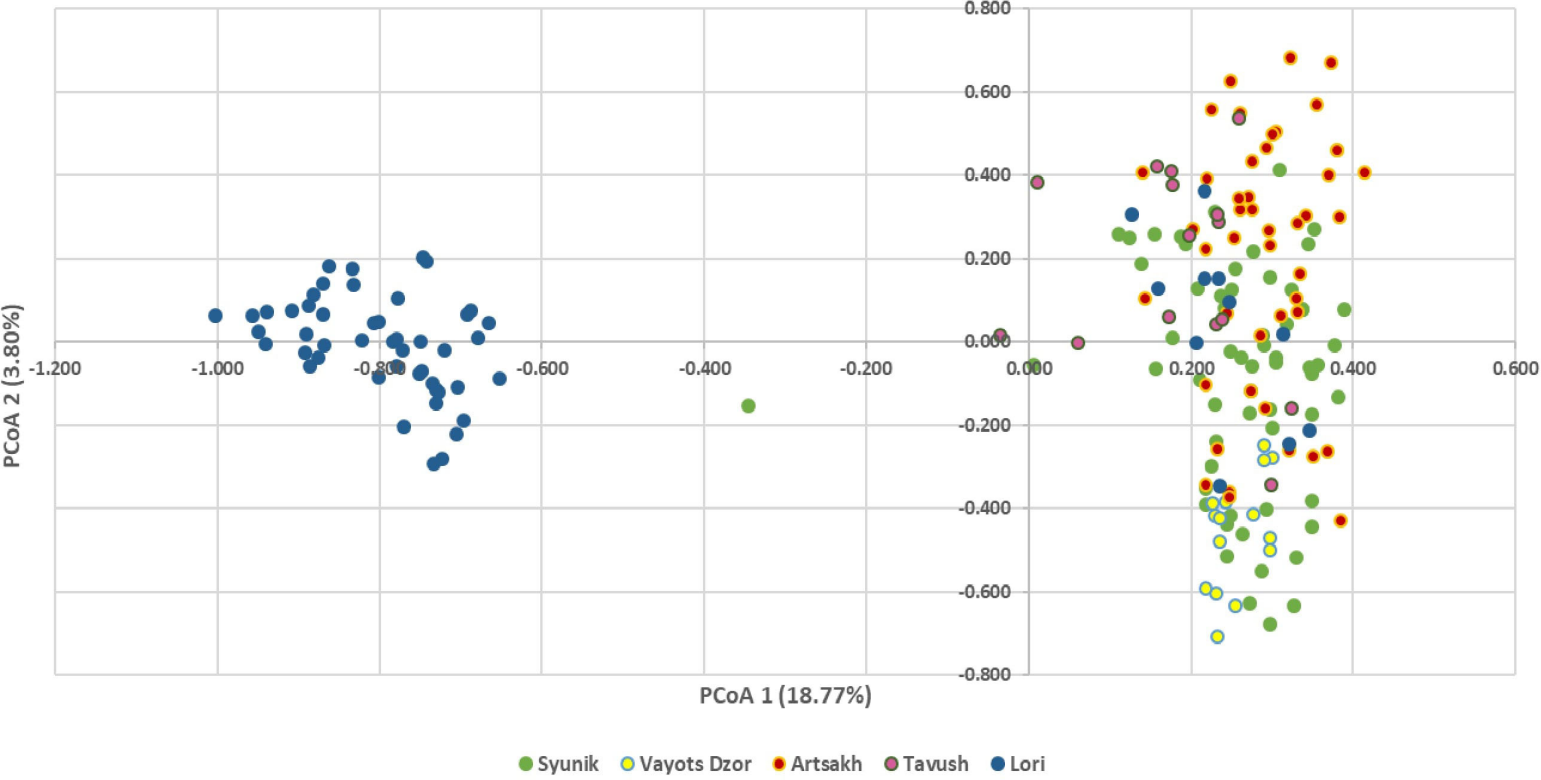
Principal coordinate analysis (PCoA) represented by two axes using a covariance matrix of 24 nSSR locus of 196 accessions. Genepool A (right) and genepool B (left) are clearly differentiated. Wild genotypes of genepool A are labeled with the corresponding color for the group to which they belong.

To identify the structure of analyzed populations and the correlations among wild genotypes from diverse geographical origins, two different analyses were performed. PCoA based on the genetic distance matrix obtained by the 24 nSSR genotype profiles revealed a similar pattern to that observed in the neighbor-joining dendrogram (**Figure 4**).

The two PCoA axes explained a total of 22.57% of the observed variance. The Alaverdi (blue) genepool B from Lori province forms an isolated gene pool on the left side of the main axis, suggesting genetic dissimilarity with genepool A collected for the present study, which groups on the right side of the main axis. Because precise information about the origin of samples included in genepool B is lacking, based on obtained results, we can hypothesize that these accessions derived from a reduced number of genetically related mother plants. The Syunik population (labeled in green) forms a sister group with the accessions from Vayots Dzor (labeled in yellow), indicating that they are genetically similar and partly also match the accessions from Artsakh (labeled in red). *V. sylvestris* from Lori and Tavush are closely associated to the right cluster. In contrast, genepool B is clearly different from samples of genepool A recently collected from Lori province. Only few wild accessions from Syunik and Tavush are placed between two main clusters.

**Figure 5:**
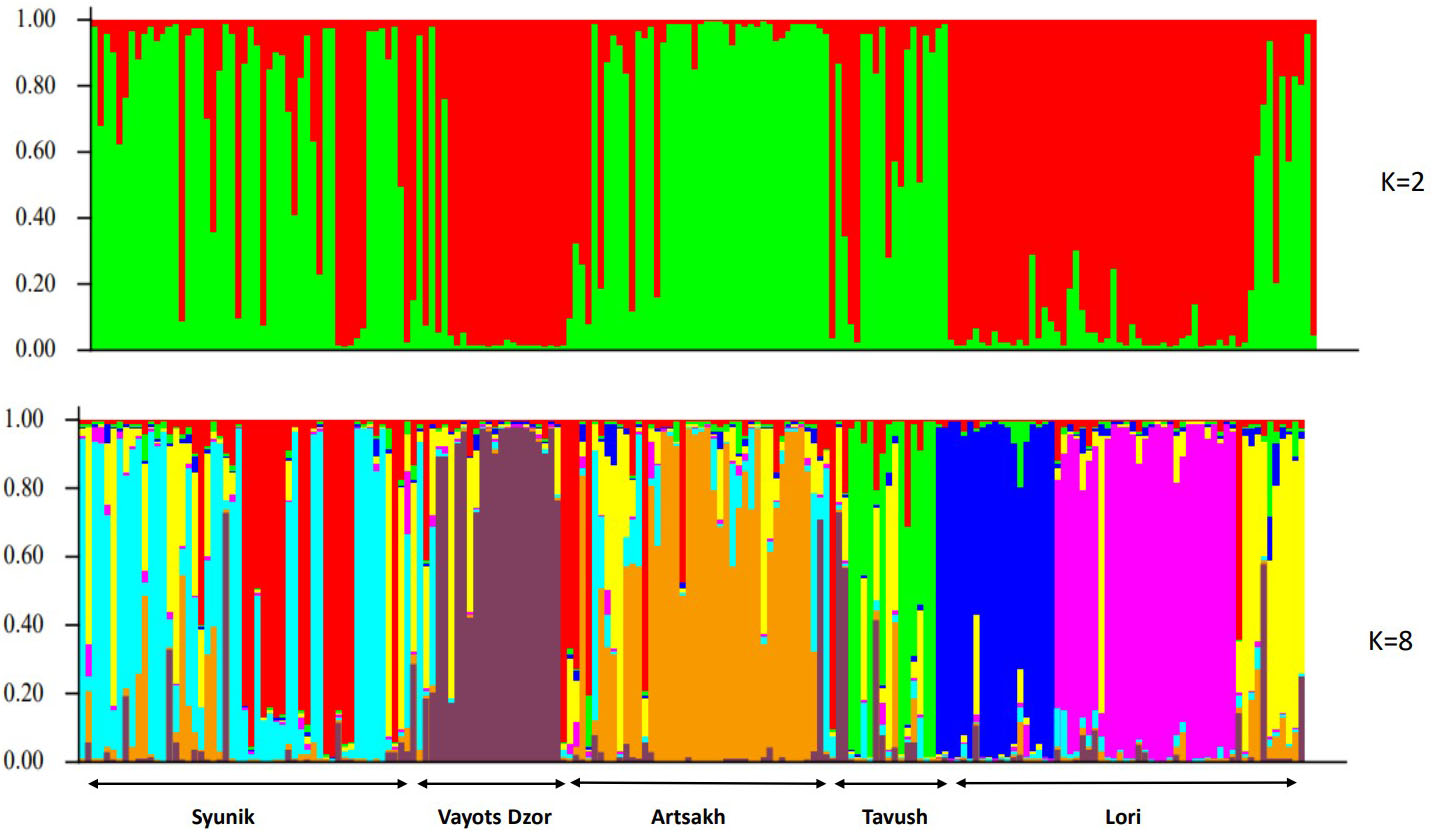
Barplot displaying the admixture proportions of 196 wild genotypes as estimated by STRUCTURE analysis at K = 2 and K=8. Each accession is represented by a single vertical bar divided into K color segments representing its proportions in the two, and eight inferred genetic clusters using STRUCTURE software. Groups were named according to the geographic locations.

The second method used to estimate genetic relationships among the 196 wild grapes from different origins was a clustering algorithm implemented in the program Structure. The results of the Bayesian analysis of genetic structure were roughly analogous to those of the NJ cluster analysis and PCoA. However, subtle population subdivisions have been clearly detected by Structure. The ΔK value suggested two possibilities, namely, K = 2 and K = 8, as the second order rate of change of likelihood distribution (**Figure 5**). The statistic of Evanno et al. (2005) showed the highest probability for K = 2 (**Supplementary Table S7**). The K = 2 structure simulation splits the wild populations into two groups in absolute correspondence to the genepool A and genepool B. Here, it is important to note that, probably, the origin of genotypes grouped in genepool B could have some impact structuring of analyzed population. Nonetheless, interpreting the value of K should be carried out with care because it provides an *ad hoc* approximation, and in some cases, population structure may be missed by STRUCTURE. Hence, we used an *ad hoc* statistic ΔK to choose the optimum number of clusters (K) based on the second-order rate of change in the log probability of data between successive K-values as proposed by Evanno et al. (2005). To gain insight into the nature of wild germplasm, further analyses of population structure beyond K = 8 are required to better understand the genetic background of these populations. With K=8 structure, the simulation differentiates the populations according to the geographic origin of the material, and at the same time, the separation of southern and northern gene pools into two main clades becomes clear.

The program also splits three sub-populations within the Lori group, where two of subpopulations belong to the genepool B and the third one have been collected within our study.

Plotting the Q matrix values as the estimated membership coefficients for each individual in each K clusters, for K = 2, disclosed clusters corresponding to their habitats. Most individuals showed an average estimated major membership proportion ≥0.70 and therefore could be classified as mainly belonging to one of the two distinct genetic groups according to their largest ancestry membership fraction (**Figure 5**). **Supplementary Table S6** presents the Q-value assignments of 196 accessions to two groups. Accessions with Q-values below 0.70 were called admixed. The proportion of admixed genotypes for the analyzed set was 5.6%.

The AMOVA analysis is presented in **Table 2**. When the total genetic variation was partitioned, 11% was attributed to the differences among populations, 8% to the differences among individuals within populations, and 81% to the differences within individuals, with levels of significance estimated over 999 permutations lower than 0.001. F_ST_, F_IS_, and F_IT_ parameters over all the loci and populations were 0.108, 0.092 and 0.190sss, respectively (p ≥ 0.001).

**Table 2:** Results of the AMOVA analysis carried out among and within five populations of wild grapevine.

Source of variation	Degree of freedom	Sum of squares	Variance components	Percentage of variation (%)	F_st_	F_is_	F_it_
Among populations	4	350.235	1.066 ***	11			
Among individuals within populations	191	1,844.622	0.812 ***	8			
Within individuals	196	1,574.500	8.033 ***	81			
Total	391	3,769.357	9.911	100	0.108 ***	0.092 ***	0.190 ***

a. The inbreeding coefficient within individuals relative to the subpopulation. b. The inbreeding coefficient within individuals relative to the total. c. The inbreeding coefficient within subpopulations relative to the total. *** p ≥ 0.001 estimated over 999 permutations.

### 3.5 Genetic polymorphism at the Ren1 locus of Armenian wild genotypes

A total of 107 *V. sylvestris* individuals from *in situ* habitats were analyzed at three SSR markers linked to the *Ren1* locus on chromosome 13 (**Supplementary Table S8**). A total of 28 wild genotypes carried three R-alleles, and 34 wild genotypes carried two R-alleles associated with PM resistance. Based on the obtained results, the Artsakh population had the most individuals carrying the three R-alleles (16), followed by Syunik (9), Lori (2), and Tavush (1). R-allele 242 at marker SC47-18 was the most frequent.

## 4 Discussion

The Armenian Highlands lies on the northern edge of Western Asia and stretches up to the Caucasus from the north. The recent story about dual domestication origin and evolution of grapevine comes to state that in Armenian Highland, human and grapevine stories are intertwined through millennia and roots of grapevine domestication are found deep in the Pleistocene, ending 11.5 thousand years ago (Dong et al., 2023). According to authors’ findings, glacial episodes split *V. sylvestris* into eastern and western ecotypes approximately 200–400 ka. The last glacial advance saw the split of the eastern ecotype into two groups that separately and parallel gave rise to a domestication process. The limited migration from the area under study is the key factor for the uniqueness of grapevine diversity preserved in Armenia. The in-depth study of this gene pool can be used to shed light on the complex grapevine history in the region.

The genus *Vitis* contains approximately 60–70 inter-fertile species, and among them was *Vitis vinifera* L., which is the species with the greatest agronomic importance. *V. vinifera* ssp. *sylvestris* is the only extant wild *Vitis* taxon native to Eurasia, and it is considered the progenitor for almost 10,000 domesticated grapevine cultivars nowadays (This et al., 2006). Wild grapes are endangered in all their distribution areas, and preservation measures are needed to maintain the genetic integrity and survival of the remnant diversity. Within this context, in the proposed study, our goal was to characterize genetic diversity via population genetics methods to decode the population signature and decipher the potential of wild grapes growing in Armenia.

Despite the importance of grapevine cultivation in human history, religion, culture, and the role of economic values of cultivar improvement, comprehensive genetic and genomic data for wild grapes in Armenia are lacking. The present work is the first study evaluating the genetic diversity and population structure of wild grapes originating from Armenia with DNA-based markers. Until now, no inventory or systematic genetic and morphological characterization of *V. sylvestris* has been carried out in Armenia. The surveys started in 2018, and thousands of plants have been registered, and this activity is an ongoing process. One of the tasks of the research group is to know the current distribution and main habitats of the fragmented populations of wild plants in the country, to carry out morphological description of *V. sylvestris* along their phenological development, to analyze its sanitary status, and to estimate genetic richness of wild plants.

### 4.1 Genetic diversity and population structure of wild grapevine in Armenia

Accurate detection and precise quantification of genetic variation is pivotal for cost-effective management and successful conservation of grapevine genetic resources. Morphological characteristics are used to distinguish wild grapes from cultivated grapes; however, in some cases, these tools do not correctly identify genotypes escaped from vineyards or hybrids between wild and cultivated plants. The application of DNA markers such as simple sequence repeats (nSSR) provides the possibility of characterizing wild accessions on the basis of their genomic signature. To characterize *Vitis* genetic resources, we have adopted a symbiotic approach as the precise strategy combining ampelography and molecular fingerprinting used in our previous studies. Ampelographic characterization of wild accessions involved in this study is ongoing. The genotyping results of the present study provides the basis to identify duplicate accessions among the sampling set and to maintain only unique genotypes and to establish a SSR marker-based database of distinct wild plants of Armenia that in the future will facilitate germplasm collection efforts.

Obtained results ensure essential information that advances the understanding of the allelic diversity, population structure of Armenian wild grapes, and hybridization patterns shown only in particular locations and in few cases. The most admix samples were observed within the Syunik population. The presence of cross-hybridization between *V. sylvestris* and *V. sativa* has been demonstrated to be a well-known phenomenon in *V. vinifera* L. (Di Vecchi-Staraz et al., 2009; De Andrés et al., 2012). The detection of naturalized hybrids in *V. sylvestris* populations is in accordance with described cases of pollen flow between vineyards and wild grapes reported by authors (Di Vecchi-Staraz et al., 2009; Riaz et al., 2018). This level of gene flow between two subspecies, occurring over many generations, could have strong effects and consequences such as introgression, pollution of the gene pool, and loss of genetic makeup on the evolution of small populations of wild grapevines (Grassi et al., 2006). Furthermore, in few cases, our results show evidence of hybridization between rootstocks and wild individuals, and these cases were only found for the Lori populations. This could be due to the existence of Phylloxera in the region since 1926, where some rootstocks and their offspring have been detected across the road and where grafted vineyards predominated. For example, among collected accessions of Millardet et Grasset 420 A, Couderc 3309 have been detected after molecular fingerprinting and comparison based on VIVC database. For some of genotypes, the hybrid alleles have been registered, like VVMD28 (252 bp), VVIP31 (269 bp), and VVS2 (123 bp).

The 24 nSSR loci analyzed displayed a different range of polymorphism, genetic diversity, and inbreeding level within the Armenian wild grape populations, as shown by the variability of the measured indices. We expected to see a high level of heterozygosity because wild grapevine species are dioecious and obligate out-crossers. According to the obtained results, the observed and expected heterozygosity in Armenian wild populations were higher than reported before for other wild populations that originated from Europe and from the Mediterranean basin (Barth et al., 2009; Lopes et al., 2009; De Andrés et al., 2012; Biagini et al., 2014), but were comparable to the results registered for the Georgian wild grapes (Imazio et al., 2013). In another manuscript by Doulati-Baneh et al. (2015), the genetic diversity of wild grapevine originated from Zagros mountains was studied. Wild grapes have been collected from five different forest locations, and based on the data, 182 alleles were detected with an average 7.9 allele per loci. The lower genetic diversity observed in their study according to the authors may be due to the small population size and the effect of random drift (Doulati-Baneh et al., 2015).

The first large-scale study performed by Margaryan et al. (2021) focused on genetic diversity and parentage analysis of 492 grapevine genotypes collected across country. The obtained high number of alleles (347 alleles, 14,485 allele/per loci), high level of observed and effective heterozygosity, and presence of female APT3-allele 366, which is absent in western European cultivars, illustrate the huge diversity of the Armenian germplasm. Authors concluded that these findings are related to recurrent introgression of *V. sylvestris* into the cultivated compartment during domestication events. Another study (data are not published) demonstrated clear distinction between cultivars and wild grapes of Armenia and showed high assignment to the *sativa* and *sylvestris* clusters. Furthermore, data also showed mixed clades or overlaps between two clusters falling in the transition zone and representing as admixed genotypes, indicating a possible common gene pool for the two subspecies. The gap related to the introgression, gene flow, and hybridization pattern between these two subspecies are still open and could be resolved after analysis of whole genome data, which is our ongoing activity. However, the obtained results indicate the high genetic diversity still preserved in the wild populations in Armenia. Previous studies based on molecular markers showed a higher wild grapevine haplotype diversity in Caucasus, which expressed the highest value, suggesting the area as a possible center of origin of the species (Grassi et al., 2006).

Evaluation of population genetic structure becomes an increasing focus providing essential insight into patterns of migration, gene flow, and demography among “populations” (Janes et al., 2017). The Structure is the most cited among several clustering-based methods, which was created to provide precise estimates without the need for populations to be determined *a priori*. However, the study by Janes et al. (2017) evidenced some facts, when the application of Structure and ΔK method introduced the problem to select “true” number of clusters, and authors concluded that many studies may have been over- or underestimating population genetic structure.

In our study, Structure analysis provided two possibilities of population clustering, namely, K = 2 and K = 8, as the second-order rate of change of likelihood distribution, which characterize more precisely the structure of Armenian wild grape populations distributed within different biogeographic regions of Armenia. The interpretation of K should be done having in mind that it provides *ad hoc* approximation and there is probability to over- or underestimate population structure. K=8 value was selected because it is biologically sensible.

The approaches used to analyze the genetic structure of *V. sylvestris* in Armenia showed that the wild population have distinct groupings and differentiations based on geographic location; at the same time, a tendency to split between the northern and southern genotypes and values of apparently dissimilar genetic indexes was recorded. Admixture analyses showed that hybridization is not high and that wild and domesticated grapevines have essentially remained reproductively isolated, most likely due to diverse relief and the geographical distance frequently observed among vineyards and mountains, forests, and riverbanks, where wild populations were mainly found.

The ampelographic characterization of wild grapes is the first important step in the conservation of *V. sylvestris* populations as an essential source of genetic diversity. Traits such as dioeciousness, color of internodes and young leaves, anthocyanin coloration on tips and buds, opening of petiole sinus, presence of teeth on sinuses, bunch and berry size, and seed shape are key traits to find and preserve wild populations in Armenia. The *in situ* and *ex situ* conservation of valuable source of wild grape diversity is essential to prevent the extinction of these unique accessions that are potentially valuable for breeding programs. Nevertheless, it would not be advisable to use only ampelography to establish relationships among wild Armenian genotypes by geographical location or gender. Some differences may be attributed to genetic adaption strategies to environmental conditions rather than genetic relationships.

**Figure 6:**
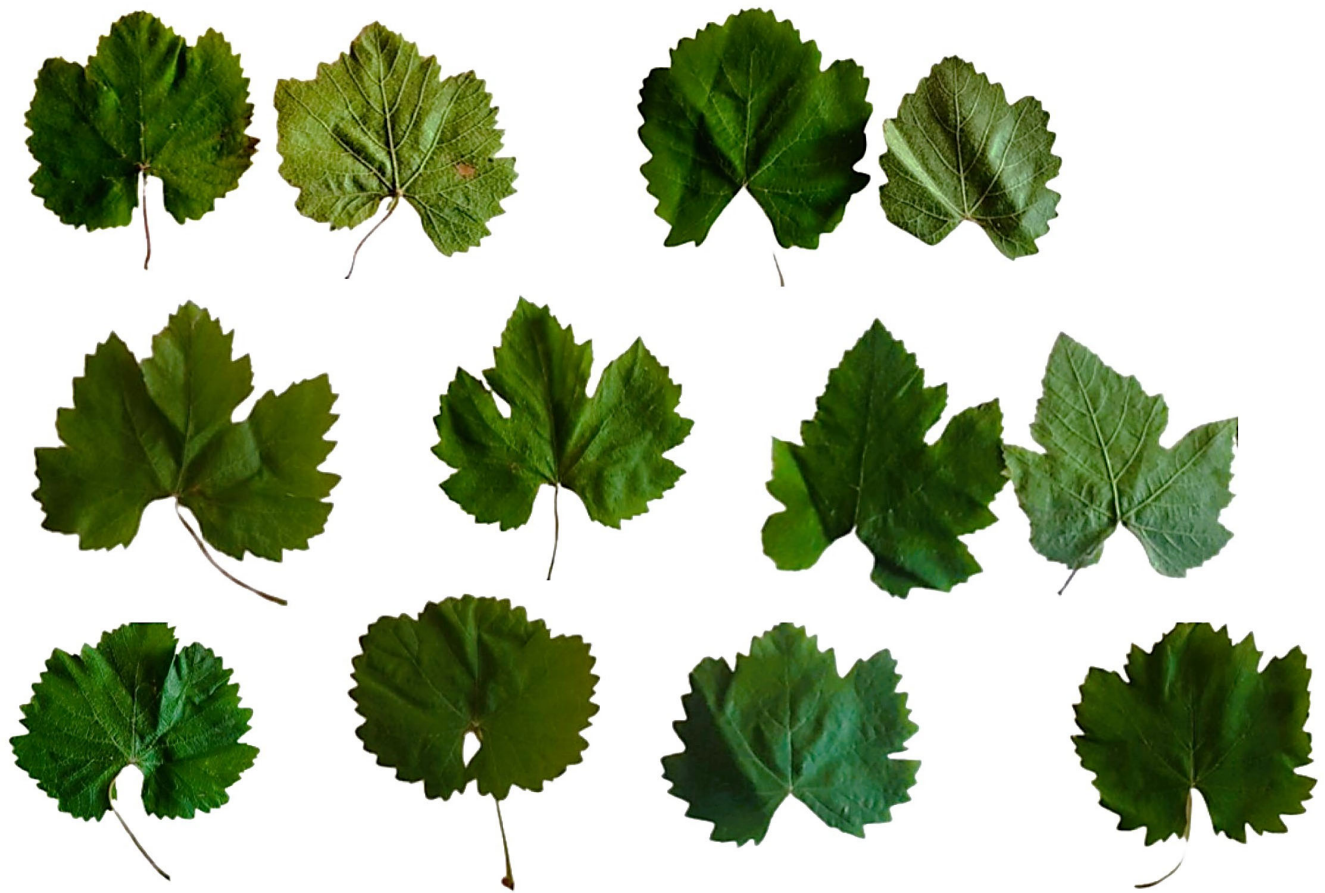
Leaf morphology of wild grapevines growing in Lori province.

Until now, no systematic genetic and morphological characterization of Armenian wild grapevine accessions has been carried out to determine whether they correspond to bona fide *V. vinifera* ssp. *sylvestris* individuals, naturalized cultivars, or spontaneous hybrids. There are only a few records, and the only detailed characterization of Armenian wild grape populations originated from Lori province, Debed River canyon, done by D.I. Sosnovskiy in 1947 in the book “Dikorastushchaya vinogradnaya loza Pambakskogo ushchelya” in Russian, translated as “Wild grape of Pambak canyon.” He described the five most important subgroups of wild plants, which show extensive morphological diversity. According to our observations the phenotypic characteristics of the leaves in Lori province are varying widely, from circular to pentagonal and wedge-shaped with three or five lobes, with petiole sinus open or just open and blistering light or moderate (**Figure 6**). Quite differently, even the pilosity of the lower side of the leaf, ranging from absent to high, was characterized by prostrate hairs, while erect hairs were detected only in a few cases in the Lori population. The variable is detected also for the color of the veins and the petiole, from light colored to the totally absent of anthocyanin coloration, which was the dominant characteristic. Describing the wild grape populations in Caucasus, Sosnovskiy (1947) concluded that only wild grapes growing in Lori region shared similar morphology with Caucasian *V. sylvestries*, while the majority of wild accessions have obvious heterogenic morphology typical of this region. At the same time, it was underlined that all these “non-canonic” wild individuals show the obvious typical characteristics of wild grapes: dioecious small sized plants, black and rounded berries, and loose or very loose bunches.

**Figure 7:**
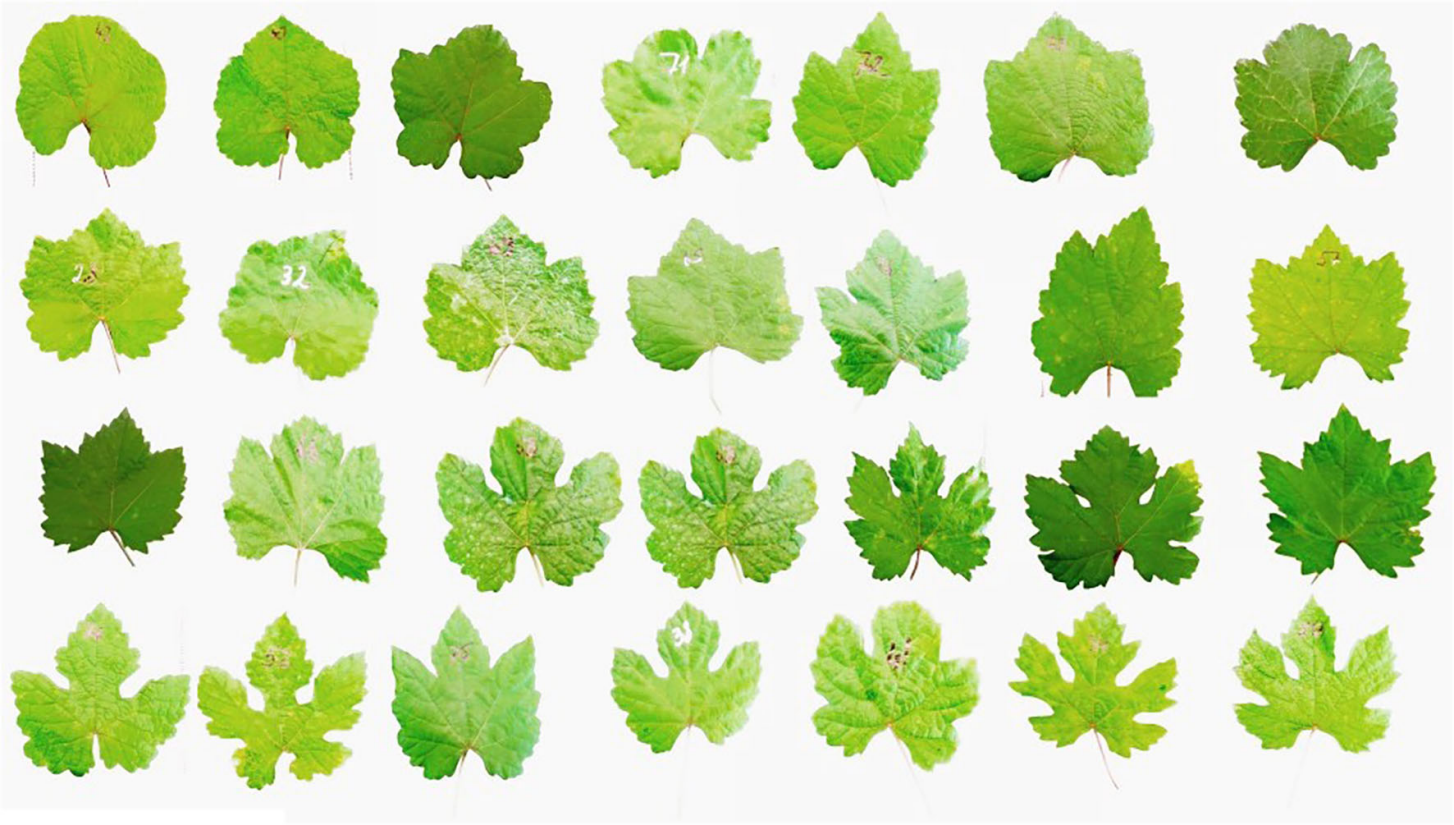
Leaf morphology of wild grapevines growing in Syunik province.

**Figure 8:**
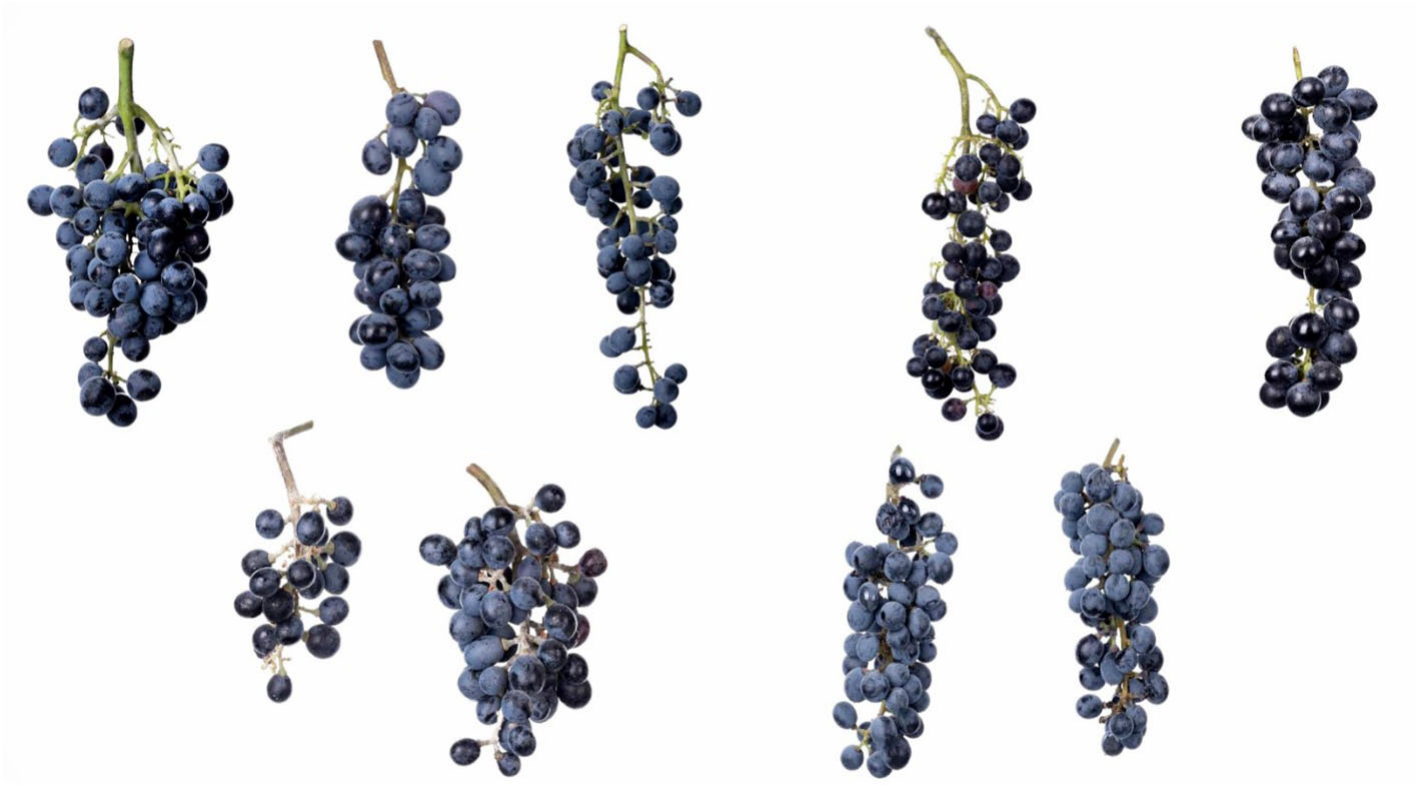
Bunch morphology of wild grapes, Syunik province.

Since 2018, the collection and characterization of wild grapes growing in Syunik and in Artsakh were also carried out. The large diversity of leaf and bunch morphology of wild grapevines growing in Syunik province are presented in **Figures 7**, **8**. In contrast to Tavush and Lori provinces, where wild grapes are growing in forests and river banks, in Syunik, Artsakh, and Vayots Dzor provinces, the wild plants are mainly growing on cliffs, climbing the rocks and in gorges.

The population of *V. sylvestris* that originated from Syunik has shown the highest genetic and also phenotypic diversity in terms of leaf, berry, and bunch morphology. The plants of Syunik province showed weaker anthocyanin coloration on shoot tips, higher frequency of bronze-colored young leaves, from wedge-shaped to five-lobed and circular mature leaves, and no teeth on the petiole sinus or in the lower and upper lateral sinus, compared to the Lori population, where teeth are presented. Other characters found to be discriminant traits, such as open petiole sinus of wild vines, loose and small bunches, small dark-blue berries with a globose (dominated shape) or ellipsoid shape, and rounded seeds.

The ellipsoid shape of berries was found only for the Syunik population. The same phenomenon is described also for Spanish wild populations, where an ellipsoid shape of berries was typical for populations from the southern part of Spain compared to the northern populations, in which spherical shaped berries are predominant (Benito et al., 2016). All these characteristics are in agreement with the results reported by other authors (Olmo, 1995; Martínez de Toda and Sancha, 1999; Barth et al., 2009; Di Vecchi-Staraz et al., 2009; Maghradze, 2022).

Nowadays, advances in both genetics and breeding programs made use of wild grapevine plants for the introgression of disease resistance genes and tolerance to diverse abiotic and biotic stresses and climate environments. Mapping genes of interest associated with agronomic and/or fruit quality traits opens up the possibility for using molecular markers for assisted selection (MAS) (De Lorenzis et al., 2022). The generated information on the domestication process and genetic resources helps to understand the gene pool available for the development of cultivars that respond to producer and consumer requirements.

In the proposed research, for the first time, we have analyzed a gene pools of wild grapes from different geographic origins and genetic backgrounds with the purpose to identify potential resistance sources against powdery mildew using three SSR markers linked to the Ren1 and Ren1.2 loci. According to the results obtained, 28 wild genotypes carried three alleles test, and for 34 of them, we have detected the presence of two R alleles providing the opportunity to eventually expand the gene pool for powdery mildew resistance breeding. The data of sequencing of resistance genes from these wild accessions would be very informative to gain further insight into the evolution of powdery mildew resistance in Armenia, where possibly powdery mildew disease existed for thousands of years and resistance in wild populations evolved over a longer time via sexual recombination. Recent studies demonstrated that the Caucasian-resistant individuals have an allelic profile different from the Ren1-carrying genotypes from Central Asia. It could be hypothesized that Eurasian *V. vinifera* grapes may have developed multiple and independent resistance genes located on chromosome 13 close to Ren1 genetic region. Summarizing all the arguments, we can conclude that PM resistance is predominant in the subsp. *sylvestris* from Armenia and neighbor countries and may have been present at the time of domestication thousands of years ago. Future studies are planned for the assessment of the structure and evolution of the underlying local resistance genes.

## 5 Conclusions

The presented study is the first extensive research forwarded on the assessment of the genetic diversity of Armenian wild grapes using nSSR markers for molecular fingerprinting. Prospections in traditional viticulture regions, forests, and mountainous areas across the country provided insights into the large diversity of *V. sylvestris* existing in the country. A combination of ampelography and nuclear microsatellite markers was argued to be valuable to determine the identity of wild plants and distinguish them from cultivars and feral types.

Population structure and genetic diversity analyses identified eight genetic groups extended through different geographic regions of the country. The genetic structure analysis of wild plants revealed groupings of population and differentiation based on geographic locations. The tendency to split between the northern and southern genotypes was observed. According to the admixture data hybridization is not high and wild and domesticated grapevines have essentially remained reproductively isolated apparently due to diverse relief. Obtained results indicated that high genetic and morphological diversity as a source of novel alleles and genotypes is still preserved in the wild populations in Armenia. Based on the preliminary screening for Ren1 resistance loci against powdery mildew, it is necessary that future in-depth studies need to bring more light into the unexploited genepool of Armenia. In conclusion, this study aims to highlight the importance of *V. sylvestris* germplasm conservation in Armenia as the unique genetic resource, which can contribute to the development of improved cultivars with enhanced disease resistance, adaptability,and quality traits.

## Data availability statement

The original contributions presented in the study are included in the article/supplementary material, further inquiries can be directed to the corresponding author/s.

## Author contributions

KM: Conceptualization, Data curation, Formal Analysis, Investigation, Methodology, Resources, Supervision, Writing – original draft, Writing – review & editing. RT: Writing – review & editing. BG: Writing – review & editing. AA: Writing – review & editing. OT: Writing – review & editing. FR: Writing – review & editing. EM: Conceptualization, Data curation, Formal Analysis, Investigation, Methodology, Writing – review & editing.
