# Supplementary figures and images for: Wild grapes of Armenia: unexplored source of genetic diversity and disease resistance
Source: Front Plant Sci. 2023 Dec 8;14:1276764. doi: 10.3389/fpls.2023.1276764 (PMC10739323; doi:10.3389/fpls.2023.1276764)

Absolute value of the 2nd order rate of change of the likelihood distribution (mean)

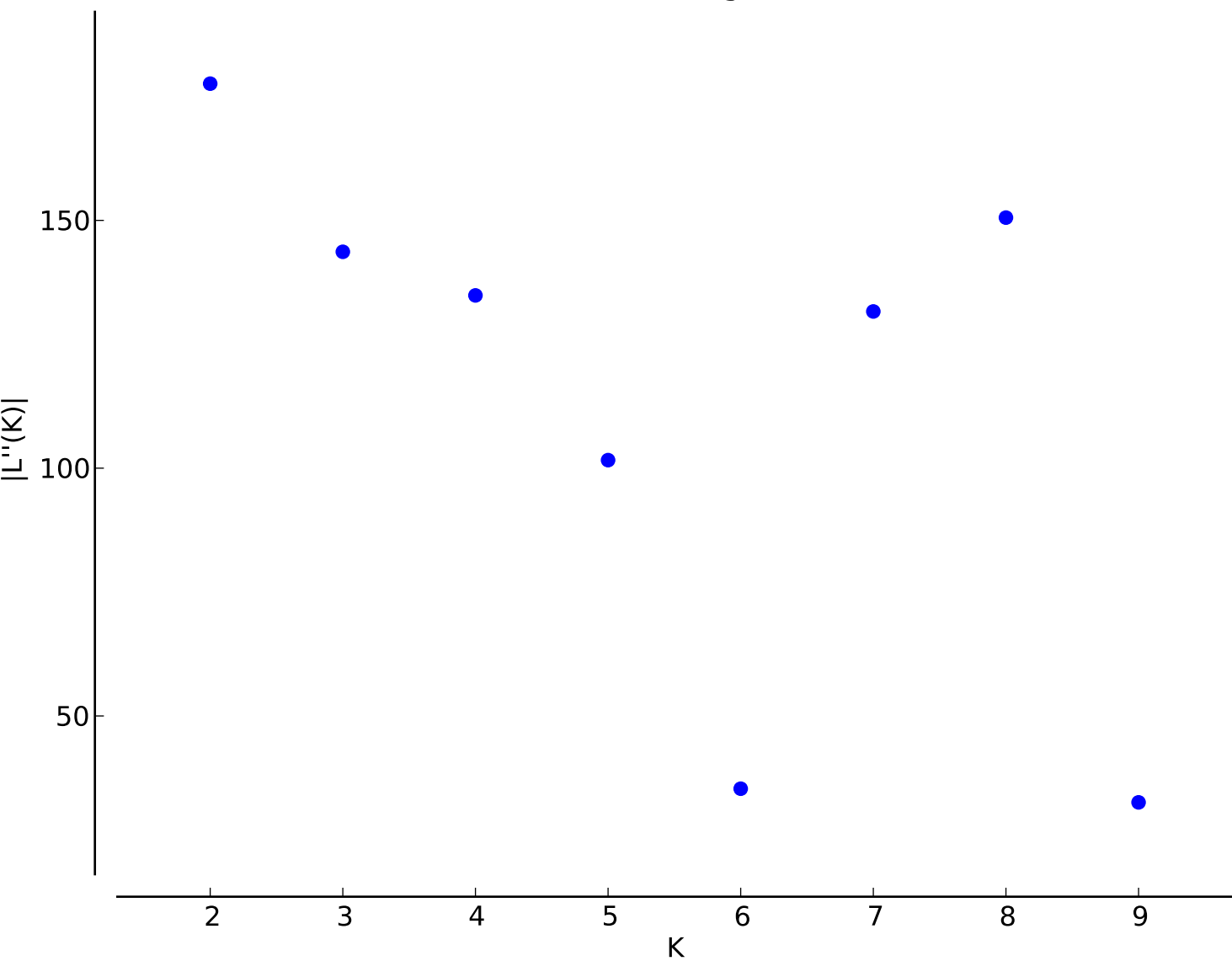

Supplement: Supplementary file 1 [file DataSheet_1.pdf]

$$\text{DeltaK} = \text{mean}(|L''(K)|) / \text{sd}(L(K))$$

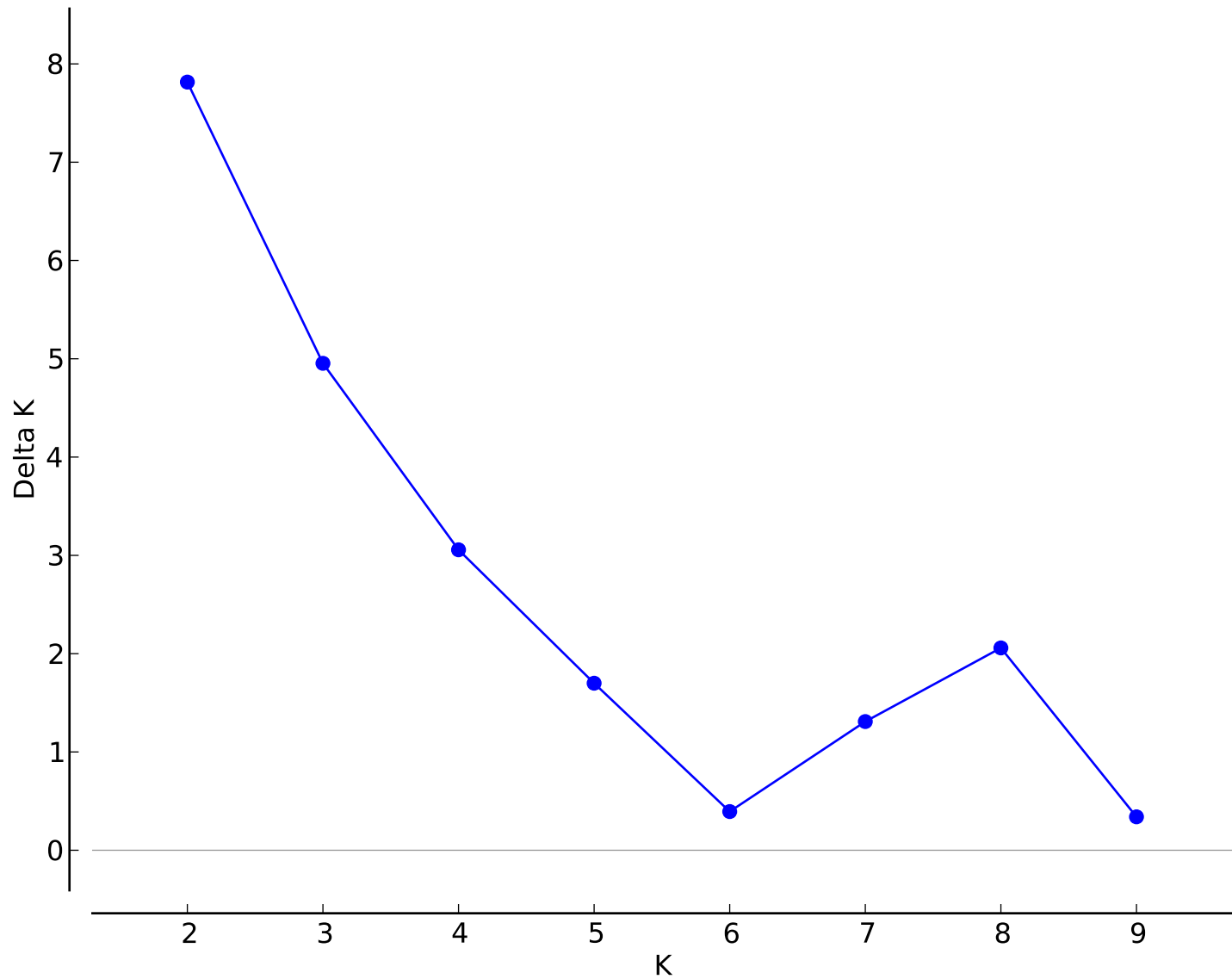

Supplement: Supplementary file 2 [file DataSheet_2.pdf]
